# Comparison of *Clostridioides difficile* Infection Incidence in a General and a Geriatric Hospital Prior to and During the COVID-19 Pandemic

**DOI:** 10.3390/jcm14134664

**Published:** 2025-07-01

**Authors:** Yochai Levy, Husam Golani, Ahmed Baya, Erica Pinco, Nira Koren, Lutzy Cojocaru, Dana Kagansky, Nadya Kagansky

**Affiliations:** 1 Shmuel Harofeh Geriatric Hospital, Beer Yaakov 70300, Israel; husam.joulani@moh.gov.il (H.G.); dr.ahmad152@outlook.com (A.B.); erica.pinco@moh.gov.il (E.P.); batia_nadya.kagansky@moh.gov.il (N.K.); 2 Faculty of Medicine, Tel Aviv University, Tel Aviv 6997801, Israel; nira.morag@gmail.com (N.K.); lutzy18@gmail.com (L.C.); danak@shamir.gov.il (D.K.); 3 Shamir (Assaf Harofeh) Medical Center, Zrifin 70300, Israel

**Keywords:** *Clostridioides difficile*, *Clostridium*, COVID-19, geriatric hospital, older adults

## Abstract

**Background**: *Clostridioides difficile* (CD) is the main cause of nosocomial diarrhea, resulting in increased morbidity and mortality, and is thought to be greatly affected by strict hygiene. In this study, we assessed changes in CD infection prevalence and outcomes pre- and during the COVID-19 pandemic (CP). **Methods**: This was an observational cohort performed at a tertiary medical center (MC) and a geriatric hospital (GH). Patients from both hospitals diagnosed with CD were included, and the period of one year prior to the pandemic to one year after was compared. Data was extracted from electronic medical records (EMR). **Results**: A total of 145 CD-associated diarrhea (CDAD) cases were diagnosed in the MC and 54 in the GH. There was no change in CDAD prevalence or mortality between the study periods in either hospital. Disease duration, measured as days with diarrhea (DWD), was shorter during the CP in the GH (10.6 days vs. 8.1 days, *p* < 0.01). CDAD was more prevalent in the GH during both periods; however, the disease was milder, with only three mortality cases and a significantly shorter disease duration (3.19 DWD vs. 10.67 in the MC before CP; 3.11 vs. 8.1 during CP, *p* < 0.01). In a survival analysis for MC patients, no significant differences were found between periods before and after adjustment for age, gender and period. **Conclusions**: The CP affected the duration but not the prevalence of CDAD. The milder course of CDAD in the GH may have been due to the quality of treatment provided in an academic GH and the subsequent faster diagnosis and treatment.

## 1. Background

*Clostridioides difficile* (CD) is one of the most common infections related to the healthcare system and results in high rates of morbidity and mortality, with substantial recurrence rates. The incidence of CD-associated diarrhea (CDAD) increased dramatically in the current millennium in the majority of countries around the world; in the United States alone, there are approximately 500,000 cases of CDAD per year and 30,000 cases of related fatalities [1].

CDAD occurs after exposure to or being a carrier of CD, usually in patients with various predisposing factors. The main CDAD risk factors are prior use of antibiotics (namely 3rd/4th generation Cephalosporins, Carbapenems, Fluoroquinolones, Clindamycin and broad-spectrum Penicillin combinations) [2,3] and poor patient and staff hygiene practices [4]. Additional risk factors include advanced age, poly-morbidity, inflammatory bowel disease and use of enteral feeding [5]. Numerous local and international guidelines have been established to improve both the prevention and treatment of the infection [6,7]. Local guidelines recommend contact isolation and provide clear instructions regarding hygiene during CDAD patient care, including the use of disposable gowns and equipment or the use of equipment reserved for use only by a single patient, handwashing protocols, training on prevention of CD transmission, and follow-up and monitoring of incidence of infection and morbidity. In recent years, the establishment and implementation of these strict guidelines have led to a trend of decreasing numbers of CD patients [8]. Bundled interventions and antimicrobial stewardship have been shown to support the reduction in CDI rates, with once- to twice-daily disinfection of high-touch surfaces and cleaning of patient rooms with chlorine-based products resulting in a 45% to 85% reduction in CDI [9]. Healthcare environmental hygiene in the hospital was also related to lower overall infection rates and/or patient colonization in a recent systematic review [10].

From 2020 to 2021, as a result of the COVID-19 pandemic (CP), an emphasis on contact and respiratory isolation was established among COVID-19 patients, and frequent trainings regarding the use of personal protective equipment (PPE) were provided to healthcare staff. These measures seemed to positively affect healthcare-associated infection in the beginning of the CP [11]. However, during this same period of time, the heavy load on the healthcare system and lack of staff served as risk factors for additional outbreaks of infections [12,13,14].

## 2. Aim

The purpose of this study was to assess the effects of the CP period upon the frequency and characteristics of morbidity with CDAD in a GH compared to a general hospital.

## 3. Methods

An observational cohort study was performed at a large medical center (MC) and a geriatric hospital (GH) in Israel. The MC is a tertiary hospital and the fourth largest in the country. The GH is a large geriatric hospital that has nine inpatient wards, including three acute geriatric wards, three complex nursing wards and two rehabilitative wards. Data was collected from EMRs in the two hospitals from one year before the CP (1 April 2019 to 31 March 2020) to one year after the beginning of the CP in Israel (1 April 2020 to 31 March 2021). Both the MC and the GH followed the same national guidelines regarding isolation measures for COVID-19 as well as for the management and prevention of *Clostridium difficile* infections, ensuring consistency in institutional hygiene protocols and antimicrobial stewardship practices.

The main outcome was the difference in CDAD prevalence between the two periods in each hospital. Secondary outcomes included number of days with diarrhea, mortality and changes observed in patient characteristics between the two periods at the MC compared to the GH. All mortality cases among patients diagnosed with CDAD were included in the analysis, regardless of the primary cause of death.

Participants: All patients that were clinically positive and tested positive for CDAD were included. CDAD was defined as diarrhea with positive confirmatory antigen testing (glutamate dehydrogenase antigen) or positive for CD toxin (toxins A and B in fecal specimens). Baseline characteristics such as age, sex, family status, place of residence, prior morbidity, chronic medications, use of antibiotics in the pre-infection period and clinical findings such as length of hospitalization, number of days with diarrhea, and mortality were extracted from EMRs.

Statistics: Data was analyzed with IBM SPSS statistics software, version 29.0 USA). Significance levels were set at 0.05. Data were assessed and described using frequency and percentages for categorical variables. Mean and standard deviation were used for continuous variables. Comparison between time periods (before and after COVID) was assessed using t-tests for independent continuous variables and chi-square tests for categorical variables. Differences between the time periods were described using OR with 95% confidence intervals. Survival between groups was described in Cox proportional hazard model curves, corrected for age, sex and time period, and correlation between different dichotomous variables was assessed.

## 4. Results

During the trial period, a total of 199 CDAD cases were diagnosed, including 145 in the MC and 54 in the GH. Table 1 describes the differences in CDAD prevalence between the two hospitals in each period. In both periods, CDAD was more prevalent in the GH. Before the COVID-19 period (CP), there were 27 CDAD patients among the 3158 total patients (0.85%) admitted to the GH compared to 76 of the 17,034 (0.45%) patients at the MC, with a significant difference between groups (*p* = 0.005). During the CP, there were 27 cases of CDAD among the 2664 total patients (1.01%) in the GH compared to 69 of the 14,375 (0.48%) total patients at the MC (*p* = 0.001). There were no significant differences between the periods in each hospital.

Several differences were found among the patients’ baseline characteristics between the two periods. In the GH group (Table 2 and Table 3) during the COVID-19 era, patients were younger (mean age of patients in Period 1 was 85.2 ± 9.3 years, compared to 78.3 ± 10.9 years in Period 2; *p* = 0.015), and fewer arrived from nursing care facilities (14.8 vs. 44.4%). There were only three fatalities during the two periods in the GH, and all three occurred prior to the outbreak of the CP. Additional significant findings included the use of aspirin, which was more common during the COVID-19 period (14% pre vs. 44% post), and increased levels of hemoglobin during the CP (10.02 vs. 11.47, *p* = 0.013). Length of hospitalization until diagnosis of CDAD was significantly longer prior to the pandemic (mean 109 days vs. 31 days, with medians of 28 vs. 15, *p* = 0.01).

An inverse correlation was found between the hospitalization rates of men and women during the time periods, which almost reached significance (*p* = 0.056); more men were hospitalized during the CP than before the pandemic, while more women were hospitalized prior to the CP. Additional trends included increased frequency of laxative use (29% vs. 11% before COVID; *p* = 0.091) and increased use of anti-depressants during the CP (37% vs. 18.5%; *p* = 0.12). There was also an increase in potassium levels (4.14 vs. 4.48; *p* = 0.056) and albumin (2.8 vs. 3.3; *p* = 0.098), while the incidence of pressure ulcers decreased during this period. There were no differences in antibiotic use between time periods.

In the MC, no differences in age, sex or place of residence were observed between time periods; however, several other significant differences were found (Table 4 and Table 5). During the pandemic, CDAD patients had shorter durations of diarrhea (mean 10.7 days vs. 8.1 days, *p* = 0.007). Other significant differences between periods in the MC included a higher incidence of anemia during the CP (75% vs. 95%; *p* < 0.001), a finding that matched the lower hemoglobin levels and lower lactate levels measured during the CP.

During the pre-pandemic era, there were 25 cases of mortality (32.9%) and 15 cases during the pandemic (21.7%), demonstrating a non-significant trend of reduced mortality during the pandemic (*p* = 0.133). Other non-significant trends observed included increased cases of hypertension and use of aspirin or any antiplatelet agents during the pandemic; however, there was a negative trend of dementia and anti-depressants use during this period of time. Significant differences in baseline characteristics and clinical findings were identified between hospitals (Table 6 and Table 7). At the GH, before COVID-19, the patients were older than those admitted to the MC, a difference that disappeared during the pandemic. During both time periods in the GH, more patients were admitted from long-term care facilities, and more patients had urinary catheters placed and had pressure ulcers. In addition, more patients received enteral feeding in the GH only during the CP. Fewer patients suffered from diabetes, anemia, cancer and pneumonia in the GH before the pandemic. Also, during that year, use of neuroleptic drugs, laxatives and anti-depressants was less prevalent in the GH. During the CP, hemoglobin levels were found to be significantly higher in the GH (*p* < 0.01).

**Figure 1:**
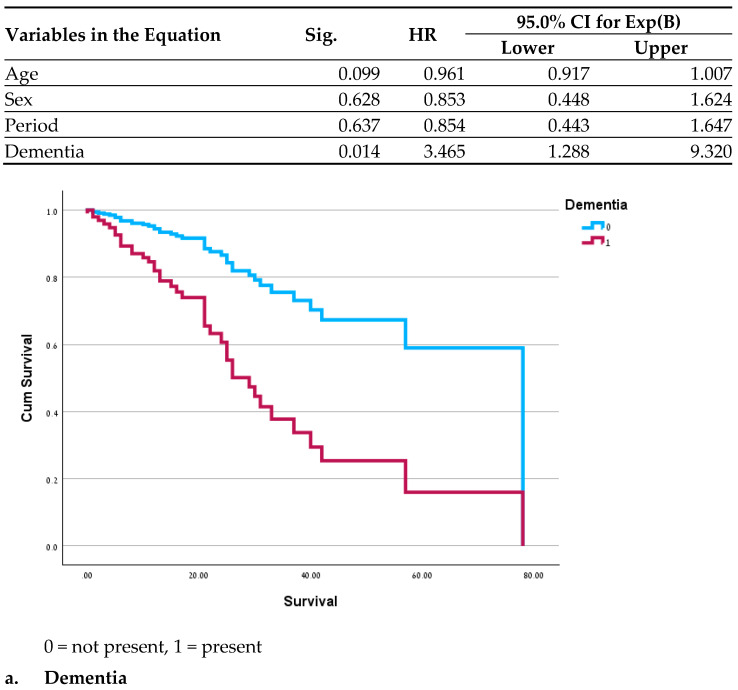

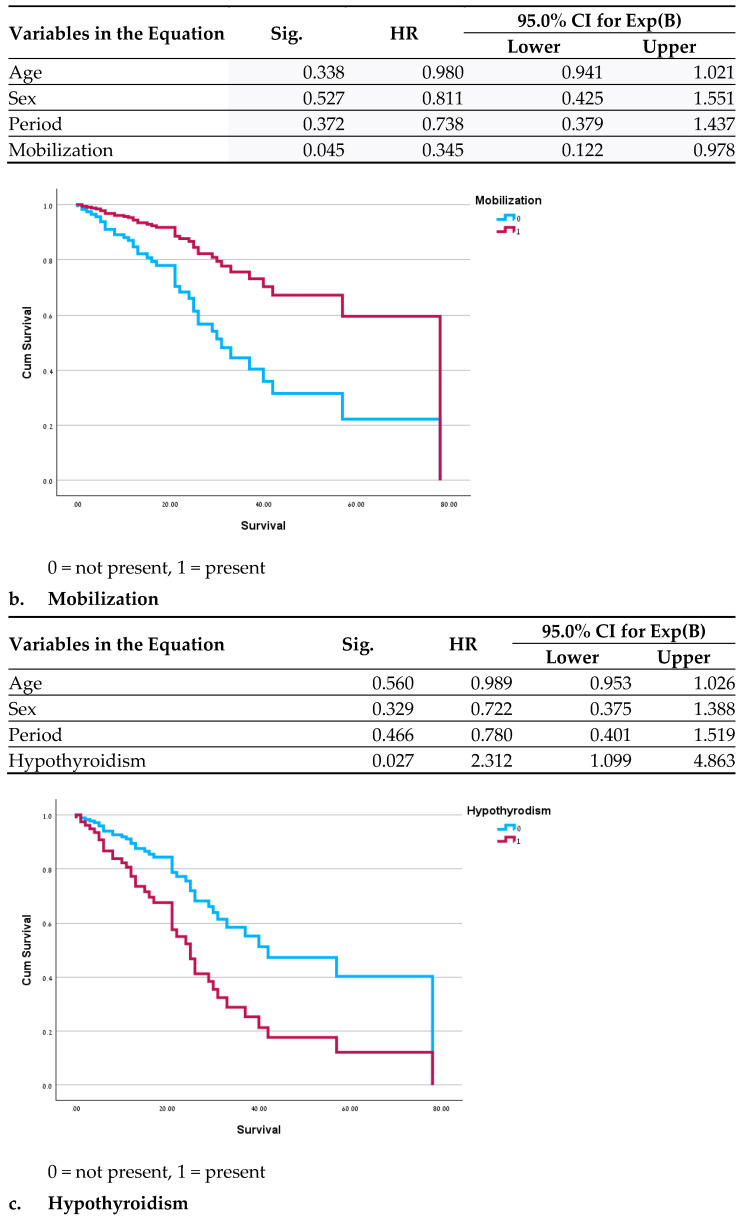

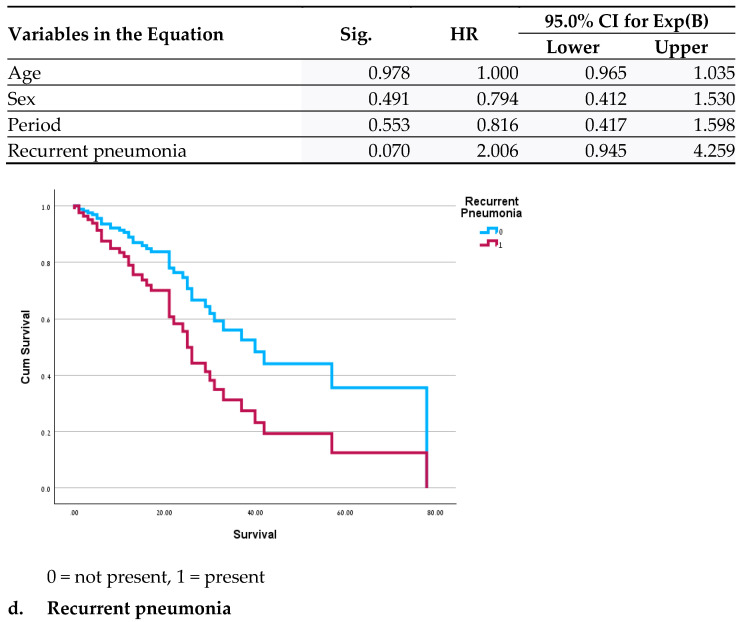
Cox Regressions adjusted for age, sex and period.

Significant differences in clinical outcomes included a lower mortality rate (*p* = 0.035 before the pandemic and 0.008 during the pandemic) and a shorter duration of diarrhea during both periods in the GH population (*p* < 0.01 for both periods). In a survival analysis for the MC patients, no significant differences were found between the two periods before and after adjustment for age, gender and period. Variables demonstrating a significant correlation with mortality were hypothyroidism (HR = 2.3; *p* = 0.027), dementia (OR = 3.4; *p* = 0.014), and reduced mobility (HR = 0.34; *p* = 0.045) (Figure 1); recurrent pneumonia almost reached statistical significance (HR = 2; *p* = 0.07). In the assessment of all the variables in a single model, no significance was found, likely due to the small sample size. Survival analysis was not performed for the GH patients due to the low mortality rate.

## 5. Discussion

In this study, we examined the correlation between CDAD before the CP and in the first year after the beginning of the CP in hospitalized older adults at a tertiary medical center compared to a GH. Our hypothesis was that strict adherence to isolation measures during the CP period would reduce cases of CDAD in both settings. No differences were found in the incidence of CDAD between the two periods in both hospitals; however, several other interesting differences were.

Data from previous studies is inconsistent regarding changes in CDAD morbidity, with few studies showing a higher incidence [15,16] and others suggesting a decline [17,18,19] during the CP. However, the majority of larger studies did not show a significant impact on CDAD during the CP [20,21,22,23,24]. These studies did not specifically address geriatric patients and mainly assessed outcomes of patients in acute settings. For example, Reveles et al. [24] examined a sample of over 22,000 CDI cases, including 12,878 pre–CP and 9261 during the CP. The median age was 68, and the vast majority of patients had an emergency admission to the hospital. A reduction in CDI was identified during the pandemic at a rate similar to that seen prior to the CP. Although the study population differed from those in prior studies (they were older, some were hospitalized in a GH, and some were from long-term care departments), no significant differences in CDI prevalence were found between the two time periods.

The differences seen in prior studies may be the result of variations in isolation methods initiated throughout the pandemic, together with hospitalization policies and COVID-19 burden in different healthcare systems around the world. Despite strict isolation policies in Israel at the beginning of the CP, no differences in CDAD prevalence before and during the CP were found in this study. It is probable that effective management of the healthcare system, which prevented the overcrowding of facilities and staff shortages, helped prevent a surge in CDAD prevalence [25]. In addition, the rigorous pre-pandemic adherence to isolation guidelines was likely already sufficient; therefore, additional isolation precautions during the pandemic did not result in lower CDAD prevalence.

Differences in the cohorts between the two time periods in each hospital point to changes in hospitalization policies and social trends, leading to differences in the consumption of medical services during the pandemic. This was more prominent in the GH during the CP, in which fewer patients were admitted from nursing homes, patients were younger, and had a lower incidence of pressure ulcers and higher albumin levels, all indicators of healthier patients. In addition, more men were admitted during the COVID-19 era, a demographic change that likely results from the higher predisposition to severe COVID-19 symptoms exhibited by males [26]. Finally, with regard to the significant elevation in aspirin treatment in both hospitals, this may be explained by the fact that cardiovascular disease is also a risk factor for severe COVID-19 [27].

In the GH, there were fewer changes during the CP compared to baseline. The main changes in this group were clinical and were expressed as fewer days of diarrhea and a trend towards lower mortality. Hemoglobin levels were lower, which may indicate the presence of more severe COVID-19 [28,29]. Possible explanations for these findings include a higher impact of PPE and adherence to guidelines, resulting in a faster diagnosis, isolation and treatment.

Differences in clinical effects were seen between the two hospitals. Despite relatively low CDAD morbidity in the two hospitals [30,31], there was a higher incidence of CDAD and shorter duration of diarrhea in the GH during both time periods. Based on prior observation, the accepted assumption is that CDAD is more prevalent in GHs and among critical patients [32]. However, these differences may also be related to under-diagnosis in long-term care facilities [32]. Skilled nursing facilities and GHs provide medical treatment to high-risk patients for CDAD infections and routinely administer oral and intravenous antibiotics to patients that often possess more risk factors [33].

Information about CDAD prevalence in skilled nursing homes is sparse and, not surprisingly, points to a high incidence rate [33,34]. Our GH is a governmental academic center that is held to strict regulatory standards and infection control monitoring. The GH was less crowded than the MC, with similar adherence to isolation guidelines and infection control. However, there were differences in treatment routines. Daily use of public areas such as common dining rooms and frequent transfers from and to bed are more common in the GH. These routines have a positive impact on cognition, function, quality of life and even mortality in older adults [35,36]; however, they may expedite CDAD infections and contribute to their higher incidence in the GH. Patients in the GH are in need of more nursing care, as can be deduced from the higher prevalence of urinary catheters, tube feeding and pressure ulcers in the GH. Furthermore, in Israel, patients admitted to skilled long-term care departments must be dependent on others for mobilization or toileting. The high need for nursing assistance may have also contributed to a greater spread of nosocomial infection.

The GH trains interns and medical personnel for comprehensive geriatric treatment of older adults. Since CDAD is seen mainly in older adults [34], the hospital medical staff has a high index of suspicion for CDAD. The facility also has in-house laboratory services that provide rapid test results for CDAD. This combination allows for fast and efficient detection and treatment of CDAD in the GH with a minimum of undiagnosed cases and may explain the high incidence, shorter disease course and very low mortality rate [37].

The more stable conditions of the CDAD patients in the GH probably also contributed to the shorter disease duration and positive outcomes. This was also suggested in laboratory markers such as albumin and hemoglobin, which were higher in the geriatric population [38,39,40,41].

It was surprising to note that the difference in age disappeared during the CP period, especially among the GH patients. The likely explanation for this shift in patient population results from the hospitalization of COVID-19 patients for the purposes of extended isolation and not due to severe illness in the GH, combined with a reduced long-term care facility patient hospitalization. Conversely, the MC was less impacted by this trend due to its size (891 versus 300 beds in the GH). Prior studies reported hospitalized patients were 1–2 years younger during the CP in general hospitals [42,43]. In this study, no significant changes were found, perhaps due to the older age of CDAD patients in both time periods, which made achieving statistical difference more challenging.

An examination of the survival curves in the MC revealed a correlation between mortality and recurring pneumonia, which may result from frequent antibiotic use among these patients. An increased mortality rate was previously reported among patients with complex conditions of both pneumonia and CDAD [44]. Similarly, dementia and level of function were also described as contributing factors to mortality and appear to indicate a more fragile health status [45,46]. We did not find a logical explanation for the effects of hypothyroidism on mortality; it may be an additional indicator of higher and more severe morbidity.

This study had several limitations related to its retrospective nature. The beginning of the CP was characterized by uncertainty and frequent changes in guidelines related to hospitalization, treatment and isolation, as well as changes in hospital resource utilization and shortages in manpower. This may have contributed to deviations in data in both hospitals. Furthermore, due to the relatively low CDAD, we could not differentiate acute departments from subacute and long-term care departments in the GH. Data on prior antibiotic use were not available for patients in the medical center (MC), which may have influenced the findings; however, the lack of significant differences in morbidity and mortality between the two periods suggests this factor had a limited impact. Moreover, the study was conducted in a single tertiary medical center and a geriatric hospital in Israel, with a relatively small sample size, which may limit the generalizability of the results to other settings or populations. We hope to expand the research and examine this topic in the future. We believe the comparison of the two hospitalization modalities presented adds important information to the current knowledge on CDAD in the geriatric population.

In conclusion, CDAD prevalence was low, without significant differences between the two periods in both hospitals, although CDAD was more prevalent in the GH. The disease was relatively mild with short duration and very low mortality. This may be an indication that current hygiene precautions against CDAD are sufficient; however, more efforts and resources should be directed to the training of skilled staff with a geriatric orientation and a high index of suspicion for CDAD. In the MC, CDAD was significantly shorter during the CP, perhaps due to a greater emphasis on infectious disease control, leading to a faster diagnosis and treatment. Differences found in the cohorts between the two eras imply that the CP resulted in significant changes to hospitalization patterns. Future research should take this into account in study designs.

## Figures and Tables

**Table 1:** Infection rate by hospital and by period.

	Before COVID-19 1.4.2019–31.3.2020	COVID-19 1.4.2020–31.3.2021	*p* (Between Periods)
MC Rate 95% C.I.	76/17,034 = 0.45% (0.30–0.50)	69/14,375 = 0.48% (0.37–0.61)	0.721
GH Rate 95% C.I.	27/3158 = 0.85% (0.56–1.24)	27/2664 = 1.01% (0.67–1.47)	0.615
*p*-value (between hospitals)	0.005	0.001	

Abbreviation: MC, medical center; GH, geriatric hospital.

**Table 2:** Comparison between the two years—geriatric hospital.

		Time Period				
		1.4.2019–31.3.2020		1.4.2020–31.3.2021		
		Count	%	Count	%	*p*
Sex	M	9	33.3%	16	59.3%	
	W	18	66.7%	11	40.7%	0.056
Death	no	23	88.5%	27	100%	
	yes	3	11.5%	0	0.0%	0.069
Place of residence	home	15	55.6%	23	85.2%	
	other	12	44.4%	4	14.8%	0.017
Use of antibiotics	no	11	40.7%	9	33.3%	
	yes	16	59.3%	18	66.7%	0.573
Co-morbidities						
Urinary catheter	no	16	59.3%	16	59.3%	
	yes	11	40.7%	11	40.7%	0.998
Pressure ulcers	no	11	40.7%	16	61.5%	
	yes	16	59.3%	10	38.5%	0.130
Feeding tube	no	21	84.0%	18	72.0%	
	yes	4	16.0%	7	28.0%	0.306
Mobilization *	1	7	25.9%	9	33.3%	
	2	11	40.7%	12	44.4%	0.640
	3	9	33.3%	6	22.2%	
Hypertension	no	2	7.4%	5	18.5%	
	yes	25	92.6%	21	77.8%	0.268
Diabetes mellitus	no	19	70.4%	13	50.0%	
	yes	8	29.6%	13	50.0%	0.123
Anemia	no	12	44.4%	18	69.2%	
	yes	15	55.6%	8	30.8%	0.069
Dementia	no	11	40.7%	13	50.0%	
	yes	16	59.3%	13	50.0%	0.498
Mild cognitive Impairment	no	19	70.4%	18	69.2%	
	yes	8	29.6%	8	30.8%	0.928
Kidney disease	no	19	70.4%	19	70.4%	
	yes	8	29.6%	8	29.6%	0.998
Hypothyroidism	no	24	88.9%	19	70.4%	
	yes	3	11.1%	8	29.6%	0.091
Heart failure	no	21	80.8%	21	77.8%	
	yes	5	19.2%	6	22.2%	0.788
Recurrent urinary infections	no	25	92.6%	26	96.3%	
	yes	2	7.4%	1	3.7%	0.552
Recurrent pneumonia	no	27	100.0%	24	88.9%	
	yes	0	0.0%	3	11.1%	0.075
Chronic medications						
Anti-psychotics	no	24	92.3%	26	96.3%	
	yes	2	7.7%	1	3.7%	0.530
Proton pump inhibitor	no	14	53.8%	10	37.0%	
	yes	12	46.2%	17	63.0%	0.219
Steroids	no	24	88.9%	26	96.3%	
	yes	3	11.1%	1	3.7%	0.299
Laxatives	no	24	88.9%	19	70.4%	
	yes	3	11.1%	8	29.6%	0.091
Anti-depressants	no	22	81.5%	17	63.0%	
	yes	5	18.5%	10	37.0%	0.129

* Mobilization: 1, independent; 2, use of a walking aid; 3, dependent on others. Abbreviation: M, men; W, women.

**Table 3:** Comparison between the two years—geriatric hospital.

	Period *	N	Mean	Std. Deviation	*p*
Age	1.00	27	85.22	9.275	
	2.00	27	78.33	10.856	0.015
Days after admission	1.00	27	109.74	150.289	
	2.00	27	31.11	38.194	0.011
Duration of diarrhea	1.00	27	3.19	2.338	
	2.00	27	3.11	2.025	0.901
Number of diseases	1.00	27	8.56	4.089	
	2.00	27	8.59	4.750	0.989
Creatinine (mg/dL)	1.00	20	1.28	0.514	
	2.00	24	1.10	0.713	0.361
Urea (mg/dL)	1.00	20	78.60	49.870	
	2.00	24	67.04	43.166	0.414
Sodium (mmol/L)	1.00	20	138.45	4.148	
	2.00	24	136.88	5.944	0.324
Potassium (mmol/L)	1.00	20	4.14	0.571	
	2.00	23	4.48	0.570	0.056
C-Reactive protein (mg/L)	1.00	17	106.65	83.415	
	2.00	18	79.56	81.771	0.339
Hemoglobin (mg/dL)	1.00	19	10.02	1.612	
	2.00	23	11.47	1.936	0.013
Albumin (g/dL)	1.00	10	2.82	0.847	
	2.00	11	3.34	0.501	0.098

Abbreviation: N, number of CDAD patients included in the analysis for each variable. * Period 1: pre-COVID-19, 1.4.2019–31.3.2020; 2: COVID-19, 1.4.2020–31.3.2021.

**Table 4:** Comparison between the two years—general hospital.

		1.4.2019–31.3.2020		1.4.2020–31.3.2021		
		Count	%	Count	%	*p*
Sex	M	36	47.4%	26	37.7%	
	W	40	52.6%	43	62.3%	0.239
Death	no	51	67.1%	54	78.3%	
	yes	25	32.9%	15	21.7%	0.133
Place of residence	home	62	81.6%	56	82.4%	
	other	14	18.4%	12	17.6%	0.904
Co-morbidities						
Urinary catheter	no	66	86.8%	60	87.0%	
	yes	10	13.2%	9	13.0%	0.984
Pressure ulcers	no	63	82.9%	56	81.2%	
	yes	13	17.1%	13	18.8%	0.786
Feeding tube	no	62	81.6%	61	88.4%	
	yes	14	18.4%	8	11.6%	0.252
Mobilization *	1	53	69.7%	51	73.9%	
	2	23	30.3%	18	26.1%	0.577
Hypertension	no	13	17.1%	6	8.7%	
	yes	63	82.9%	63	91.3%	0.134
Diabetes mellitus	no	36	47.4%	31	44.9%	
	yes	40	52.6%	38	55.1%	0.768
Anemia	no	19	25.0%	3	4.3%	
	yes	57	75.0%	66	95.7%	<0.01
Dementia	no	28	36.8%	33	47.8%	
	yes	48	63.2%	36	52.2%	0.181
Hypothyroidism	no	63	82.9%	56	81.2%	
	yes	13	17.1%	13	18.8%	0.786
History of cancer	no	55	72.4%	48	69.6%	
	yes	21	27.6%	21	30.4%	0.710
Heart failure	no	40	52.6%	36	52.2%	
	yes	36	47.4%	33	47.8%	0.956
Recurrent urinary infections	no	69	90.8%	63	91.3%	
	yes	7	9.2%	6	8.7%	0.914
Recurrent pneumonia	no	64	84.2%	64	92.8%	
	yes	12	15.8%	5	7.2%	0.110
Long-term medications						
Anti-psychotics	no	56	73.7%	56	81.2%	
	yes	20	26.3%	13	18.8%	0.284
PPIs	no	32	42.1%	24	34.8%	
	yes	44	57.9%	45	65.2%	0.366
Steroids	no	64	84.2%	60	87.0%	
	yes	12	15.8%	9	13.0%	0.639
Laxatives	no	55	72.4%	52	75.4%	
	yes	21	27.6%	17	24.6%	0.682
Anti-depressants	no	43	56.6%	47	68.1%	
	yes	33	43.4%	22	31.9%	0.153

* Mobilization: 1, independent; 2, dependent on others. Abbreviation: M, men; W, women.

**Table 5:** Comparison between the two years—general hospital.

	Period *	N	Mean	Std. Deviation	*p*
Age (years)	1.00	76	81.18	9.043	
	2.00	69	80.49	8.222	0.632
Duration of diarrhea (days)	1.00	60	10.67	5.686	
	2.00	67	8.12	4.804	<0.01
Number of diseases	1.00	76	9.63	2.934	
	2.00	69	9.97	2.875	0.484
Creatinine (mg/dL)	1.00	76	1.6250	1.37901	
	2.00	69	1.7545	1.45494	0.583
Urea (mg/dL)	1.00	76	82.826	69.9739	
	2.00	69	95.741	70.9277	0.272
Sodium (mmol/L)	1.00	76	138.213	6.9870	
	2.00	69	137.142	8.5247	0.408
Potassium (mmol/L)	1.00	76	4.0305	0.72903	
	2.00	69	4.2213	0.80952	0.138
C-Reactive protein (mg/L)	1.00	73	83.2518	76.83666	
	2.00	69	128.0800	245.97334	0.140
Hemoglobin (mg/dL)	1.00	76	10.941	2.1167	
	2.00	69	9.996	2.3776	0.012
White blood cells (k/uL)	1.00	76	11.593	6.2325	
	2.00	69	12.113	6.4972	0.624
Lactate (mg/dL)	1.00	35	2.72	2.325	
	2.00	38	1.56	0.811	<0.01
Albumin (g/dL)	1.00	75	28.8324	5.44639	
	2.00	68	28.7909	5.39850	0.964

Abbreviation: N, number of CDAD patients included in the analysis for each variable. * Period 1: pre-COVID-19, 1.4.2019–31.3.2020; 2: COVID-19, 1.4.2020–31.3.2021.

**Table 6:** Comparison between the two hospitals.

		1.4.2019–31.3.2020					1.4.2020–31.3.2021				
		Shamir		Shmuel			Shamir		Shmuel		
		N	%	N	%	*p*	N	%	N	%	*p*
Sex	W	36	47%	18	67%		26	38%	11	41%	
	M	40	53%	9	33%	0.116	43	62%	16	59%	0.818
Death	0	51	67.1%	23	88.5%		54	78.3%	27	100.0%	
	1	25	32.9%	3	11.5%	0.035	15	21.7%	0	0.0%	<0.01
Place of Residence	0	62	81.6%	15	55.6%		56	82.4%	23	85.2%	
	1	14	18.4%	12	44.4%	<0.01	12	17.6%	4	14.8%	0.739
Urinary catheter	0	66	86.8%	16	59.3%		60	87.0%	16	59.3%	
	1	10	13.2%	11	40.7%	<0.01	9	13.0%	11	40.7%	<0.01
Pressure ulcers	0	63	82.9%	11	40.7%		56	81.2%	16	61.5%	
	1	13	17.1%	16	59.3%	<0.01	13	18.8%	10	38.5%	0.047
Feeding tube	0	62	81.6%	21	84.0%		61	88.4%	18	72.0%	
	1	14	18.4%	4	16.0%	0.784	8	11.6%	7	28.0%	0.055
Hypertension	0	13	17.1%	2	7.4%		6	8.7%	5	18.5%	
	1	63	82.9%	25	92.6%	0.220	63	91.3%	21	77.8%	0.100
Diabetes (type 2)	0	36	47.4%	19	70.4%		31	44.9%	13	50.0%	
	1	40	52.6%	8	29.6%	0.040	38	55.1%	13	50.0%	0.658
Anemia	0	19	25.0%	12	44.4%		3	4.3%	18	69.2%	
	1	57	75.0%	15	55.6%	0.058	66	95.7%	8	30.8%	<0.01
Dementia	0	28	36.8%	11	40.7%		33	47.8%	13	50.0%	
	1	48	63.2%	16	59.3%	0.720	36	52.2%	13	50.0%	0.850
Hypothyroidism	0	63	82.9%	24	88.9%		56	81.2%	19	70.4%	
	1	13	17.1%	3	11.1%	0.460	13	18.8%	8	29.6%	0.250
Heart failure	0	40	52.6%	21	80.8%		36	52.2%	21	77.8%	
	1	36	47.4%	5	19.2%	0.012	33	47.8%	6	22.2%	0.022
Recurrent urinary infections	0	69	90.8%	25	92.6%		63	91.3%	26	96.3%	
	1	7	9.2%	2	7.4%	0.776	6	8.7%	1	3.7%	0.398
Recurrent pneumonia	0	64	84.2%	27	100%		64	92.8%	24	88.9%	
	1	12	15.8%	0	0.0%	0.028	5	7.2%	3	11.1%	0.538
Antipsychotics	0	56	73.7%	24	92.3%		56	81.2%	26	96.3%	
	1	20	26.3%	2	7.7%	0.046	13	18.8%	1	3.7%	0.059
Proton pump inhibitors	0	32	42.1%	14	53.8%		24	34.8%	10	37.0%	
	1	44	57.9%	12	46.2%	0.299	45	65.2%	17	63.0%	0.835
Steroids	0	64	84.2%	24	88.9%		60	87.0%	26	96.3%	
	1	12	15.8%	3	11.1%	0.554	9	13.0%	1	3.7%	0.178
Laxatives	0	55	72.4%	24	88.9%		52	75.4%	19	70.4%	
	1	21	27.6%	3	11.1%	0.081	17	24.6%	8	29.6%	0.616
Anti-depressants	0	43	56.6%	22	81.5%		47	68.1%	17	63.0%	
	1	33	43.4%	5	18.5%	0.021	22	31.9%	10	37.0%	0.630

Abbreviation: N, number of CDAD patients included in the analysis for each variable;0 = no; 1 = yes, except place of residence 0 = home, 1 = long term care.

**Table 7:** Comparison between the two hospitals.

		1.4.2019–31.3.2020				1.4.2020–31.3.2021				
	Hosp	N	Mean	SD	*p*	Hosp	N	Mean	SD	*p*
Age (years)	GH	76	81.18	9.043		GH	69	80.49	8.222	
	MC	27	85.22	9.275	0.050	MC	27	78.33	10.856	0.295
Duration of diarrhea	GH	60	10.67	5.686		GH	67	8.12	4.804	
	MC	27	3.19	2.338	<0.01	MC	27	3.11	2.025	<0.01
Number of diseases	GH	76	9.63	2.934		GH	69	9.97	2.875	
	MC	27	8.56	4.089	0.145	MC	27	8.59	4.750	0.168
Creatinine (mg/dL)	GH	76	1.6250	1.37901		GH	69	1.754	1.454	
	MC	20	1.2790	0.51396	0.080	MC	24	1.102	0.7130	0.038
Urea (mg/dL)	GH	76	82.826	69.9739		GH	69	95.741	70.92	
	MC	20	78.600	49.8697	0.801	MC	24	67.042	43.16	0.022
C-Reactive protein (mg/L)	GH	73	83.251	76.836		GH	69	128.08	245.9	
	MC	17	106.64	83.415	0.269	MC	18	79.555	81.77	0.413
Hemoglobin (mg/dL)	GH	76	10.941	2.1167		GH	69	9.996	2.377	
	MC	19	10.021	1.6120	0.080	MC	23	11.470	1.935	<0.01

Abbreviation: GH, geriatric hospital; MC, medical center.

## Data Availability

Data available on request due to ethical restrictions.
